# Aberrant overexpression of transcription factor Forkhead box D1 predicts poor prognosis and promotes cancer progression in HNSCC

**DOI:** 10.1186/s12885-021-08868-4

**Published:** 2021-11-12

**Authors:** Jin Li, Tingyuan Yan, Xiang Wu, Xueping Ke, Xin Li, Yumin Zhu, Jianrong Yang, Zhongwu Li

**Affiliations:** 1 Department of Oral and Maxillofacial Surgery, The Affiliated Stomatological Hospital of Nanjing Medical University, 136 Hanzhong Road, Nanjing, 210029 Jiangsu People’s Republic of China; 2 Jiangsu Key Laboratory of Oral Diseases, Nanjing Medical University, Nanjing, 210029 Jiangsu China; 3 Jiangsu Province Engineering Research Canter of Stomatological Translation Medicine, Nanjing, Jiangsu China

**Keywords:** Head and neck squamous cell carcinoma (HNSCC), Forkhead box D1(FOXD1), Proliferation, Migration, Invasion

## Abstract

**Objectives:**

Forkhead box D1, the core transcription factor member of FOX family, has gradually seen as a key cancerous regulatory. However, its expression and carcinogenicity in head and neck squamous cell carcinoma (HNSCC) have not been reported yet. This study was to investigate its expression pattern, clinicopathological significance and biological roles in HNSCC.

**Methods:**

HNSCC data from The Cancer Genome Atlas (TCGA) and Gene Expression Omnibus (GEO) was used to indicate the detailed expression pattern and outcome association of FOXD1, while Western Blot assay to detect FOXD1 level in a panel of HNSCC cell lines as well as immunocytochemistry to explore FOXD1 protein abundance and sublocation. Series of siRNA-mediated FOXD1 knock-down experiments to assess the proliferation, migration, invasion and anti- apoptosis ability after FOXD1 down-regulation. Bioinformatic analysis to find out which biological function and cancer-related pathways of FOXD1 associated genes involved in.

**Results:**

FOXD1 mRNA was significantly overexpressed in TCGA-HNSCC, GSE6631, GSE12452, GSE25099 and GSE30784. Besides, IHC results shown that nuclear location FOXD1 protein was significantly higher in primary HNSCC specimens from cohort involved in this study. Also, FOXD1 abundance was significantly correlated with cervical node metastasis and poor over-all/disease-free survival after combination analysis with patient pathological information. siRNA-mediated FOXD1 knock-down significantly inhibited cell proliferation, migration and invasion and induced apoptosis in HNSCC cells. Further analysis of GSEA, GO and KEGG showed that FOXD1 expression was significantly associated with oncological function and cancer-related pathways.

**Conclusions:**

Taken together, our study implies that the potential oncogene, FOXD1, facilitates oncological behavior who can be identified as a brand-new HNSCC biomarker with diagnostic and prognostic significance.

**Supplementary Information:**

The online version contains supplementary material available at 10.1186/s12885-021-08868-4.

## Introduction

Malignant tumor in head and neck mainly occurrences in oral cavity, nasal cavity, sinus, throat and pharynx, which pathological type is mainly squamous cell carcinoma (except thyroid tumor). It is the sixth most common malignant tumor in developed countries [1, 2]. According to the Global Cancer Statistics, there are over 550,000 new HNSCC patients and 300,000 HNSCC deaths worldwide every year [1, 3]. The main inducers of HNSCC include environmental risk, virus infection and gene mutation. Although clinical treatments of HNSCC patients has significantly been improved and improved the patient’s quality of life over the past decades, the prognosis of HNSCC is still unsatisfying, with the 5-year survival rate remaining around 50–60% [2, 3]. Therefore, exploring the potential molecular mechanisms of HNSCC will contribute to the development of molecular targeted therapies for this dreaded disease.

Forkhead box D1 (FOXD1), the transcription factor member of FOX family, mainly located on 5q13.2 (hg38.p13), encoding a vital DNA-binding protein containing 100 amino acids [4]. Numerous studies thus far have shown that FOXD1 as a key role in the occurrence, development and malignant regulation of multitudinous human malignance and its abnormal up-regulation was obviously related to cell proliferation, tumor metastasis and invasion as well as poor prognosis [5, 6]. However, FOXD1 function in the development of tumor biology and related molecular mechanism is still in preliminary exploration stage. Abnormal overexpression of FOXD1 gene may be as an oncogene, via facilitating cell proliferation and transformation, promoting the process of EMT and inhibiting cell apoptosis [7–9]. In addition, FOXD1 is further proved to regulate the cancer stem cells properties including tumor initiation, chemotherapy resistance and self-renewal capacities on mesenchymal glioma cells [10]. Moreover, FOXD1 can promote tumor progression and therapeutic resistance by inhibiting P27 mRNA expression in breast cancer [11].

All of the above results indicate that FOXD1 is probably a variety of new potential cancer-causing genes in human cancer. Although FOXD1 is thought to be a key oncogene in tumorigenesis, whether it takes an pivotal part in the carcinogenesis of HNSCC remains unknown. Therefore, in the present study, we collected relevant HNSCC materials from the clinical specimens, TCGA and GEO databases and executed the current systematic analysis to study the potential molecular regulatory mechanisms and clinical significance of FOXD1 in HNSCC. At the same time, the biological function of FOXD1 in HNSCC was further explored by combining small interference RNA and bioinformatics analysis techniques.

## Materials and methods

### Cell lines

A panel of HNSCC cell lines including Cal27, Fadu, SCC9, SCC25, HN4, HN6 and human immortalized oral mucosa keratinocytes (HOK) were used in this study. HOK, Cal27, Fadu, SCC4 and SCC25 cells were purchased from ATCC (USA). Specifically, two HNSCC cell lines HN4 and HN6 were gifted from Dr. Wantao Chen (SJTU, Shanghai, PRC). All cells lines were routinely tested as mycoplasma-free. Cells were grown in DMEM/F12 (Gibco, USA) supplemented with 10% fetal bovine serum (Gibco, USA) and penicillin streptomycin (1%, Beyotime, PRC), and culturaled under the condition of 37 °C (with 5% CO_2_). All regents were purchased from Sigma-Aldrich unless otherwise stated.

### Small interference RNA (siRNA)

Two independent siRNA targeting h-FOXD1 mRNA region (NM_004472.3) were obtained from GenePharma (Shanghai, PRC). The detailed sequence as follows: siFOXD1–1:GAGCACUGAGAUGUCCGAUTT; siFOXD1–2:GGAAACAGACAUCGACGUGTT; siNC: TTCTCCGAACGTGTCACGT. 100 nM of siRNAs were transiently transfected into cells with Lipo-8000 (Beyotime, PRC), and harvested for further analysis at 48 h later.

### Cell migration and invasion assay

In vitro experiments on cell migration were assessed by wound healing assay (experimental test time points: 0 h, 6 h, 12 h). Invasion experiments were carried on 24 aperture Transwell plank (8 μm, Conning, USA) with Corning Matrigel (BD, USA) (experimental test time points: 26 h). The detailed steps of two assays we all had previously reported [12].

### Cell proliferation assay and apoptosis assay

Cell proliferation/viability was detected by CCK-8 cell viability assay (Beyotime, PRC). CCK-8 solution (10 μl per well) was added at specified time-points, incubating for 2 h and tested. Apoptosis cell were detected by FACs. Cells were treated with EDTA-free-trypsin (Gbico, USA), counted, incubated with Annexin V/PI Kit (BD, USA) and tested.

### RNA extraction and qRT-PCR

Total RNA was extracted from cells with TRIzol reagent (Invitrogen, USA), reverse transcription to cDNA and using PrimeScriptTM RT-PCR kit (Takara, JP) for quantitative Real-time PCR. Relative mRNA expression was quantified as compared to internal control h-GAPDH using ΔCT method. The primers were listed as follows: h-FOXD1 forward, 5′-GATCTGTGAGTTCATCAGCGGC-3′ and reverse,5′-TGACGAAGCAGTCGTT GAGCGA-3′. h-N-cadherin forward,5′-CAAGATGGGTCAATGGAAATAG-3′ and reverse, 5′-CTCAGGAATACGAGCCTTCAC-3′; h-E-cadherin forward, 5′-AAGACAA AGAAGGCA AGGT-3’and reverse, 5′-AGAGAGTGTATGTGGCAATG-3′; h-Vimentin forward, 5′-CGAGGAGAGCAGGATTTCTC-3′ and reverse,5′-GGTATCAACCAGA GGGAGTGA-3′. h-SNAI1 (Snail) forward, 5′-CCTCCCTGTCAGATGAGGAC-3′ and reverse, 5′-CCAGGCTGAGGTATTCCTTG-3′. h-GAPDH forward, 5′-AGGTGAAGGTCGGA GTCAAC-3′ and reverse, 5′-AGTTGAGGTCAATGAAGGGG-3′.

### Western blot analysis

The total protein of the cells was extracted by Cell lysis buffer for Western and IP (Beyotime, PRC) which contains with 1% PMSF (Beyotime, PRC). Protein samples were loaded and separated by 8–12% SDS-PAGE and transferred to 0.45 μm-PVDF membranes (Millipore, USA) and blocking by QuickBlock™ Blocking Buffer for Western Blot (Beyotime, PRC). These blots were incubated at 4 °C overnight with primary antibodies against FOXD1 (1:800 dilution, ab129324, Abcam, USA), E-cadherin (1:1000 dilution, #3195, CST), Vimentin(1:1000 dilution, #2118, CST), N-cadherin(1:1000 dilution, #13116, CST) and GAPDH (1:2000 dilution, #2118,CST) followed by incubations with the corresponding secondary antibodies.

### Bioinformatics analysis of HNSCC

FOXD1 level and associated clinical data of HNSCC were downloaded from 2 publicly datasets: GEO (https://www.ncbi.nlm.nih.gov/gds/) and TCGA (https://cancergenome.nih.gov/). Specifically, R packages TCGAbiolinks was used for automatically sorting and normalizing the raw expression/clinical data of TCGA-HNSC. The overall and disease-free survival plot were downloaded from GEPIA2(http://gepia2.cancer-pku.cn/). FOXD1 correlated-gene chart (.tsv) was obtained from cBioPortal(https://www.cbioportal.org/), and genes with Spearman’s correlation > 0.2 was filtered for next step analysis. Median-cutoff was used to definition the patients from TCGA-HNSC cohort who has the high/low FOXD1 expression, then differential display analysis of gene expression between these two groups was performed by R packages limma. GSEA, GO and KEGG analysis was analysis and performed on R packages ClusterProfiler [13].

### Clinical samples and immunohistochemical staining (IHC)

This study protocol was approved by the Research Ethic Committee of Nanjing Medical University. A retrospective cohort of a total number of 110 patients with primary HNSCC treated at our institution from Jan. 2011 to Dec. 2016 were enrolled. The set of standards for: no pre-treated (including tumor resection and neck dissection surgery, chemotherapy or radiotherapy) primary HNSCC patients, detailed information available including epidemiologic, clinical, pathological, follow-up data and written informed consent from these patients. We named this cohort as “NS20 cohort”. The archived tissue samples were retrieved and used for immunohistochemical staining. IHC score was qualified by senior pathologists dependently. 13 histologically confirmed normal oral mucosa samples from non-cancer surgeries were also enrolled. The detailed steps of IHC we had reported previously [14].

### Statistical analyses

Data analysis was performed on GraphPad Prism 8.0.1 or R packages ggplot2. Normally distributed data were analyzed using an unpaired Student’s *t* test (two-tailed); multiple comparisons used nonparametric one-way ANOVA. Median cutoff and Log-rank (Mantel-Cox) test was used for survival Kapler-Merier plot. Statistical significance was defined as *P < 0.05 and **P < 0.01.

## Results

### FOXD1 mRNA is obviously up-regulated in public HNSCC sets

**Fig. 1:**
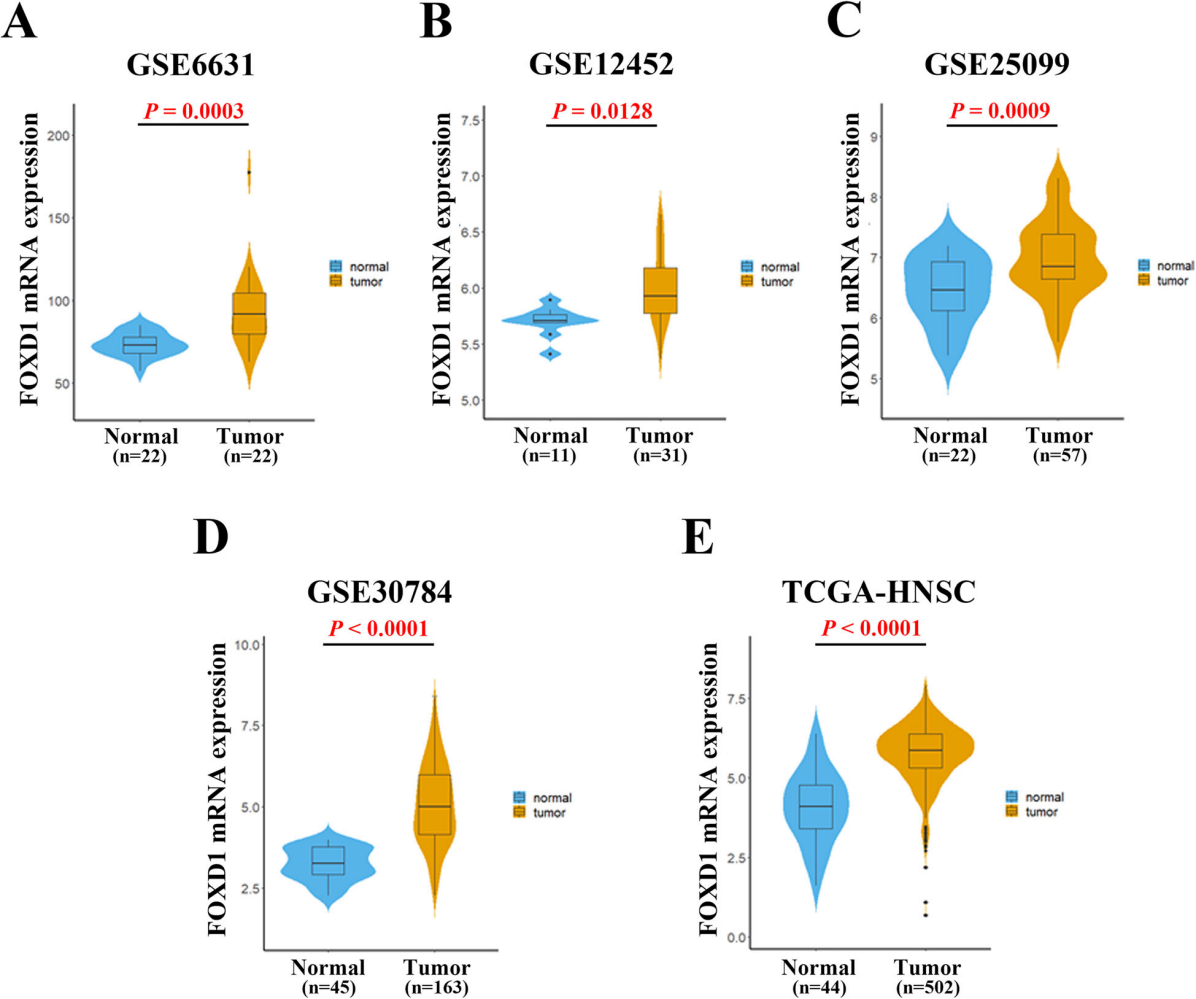
Overexpression of FOXD1 mRNA in five HNSCC datasets. **A-E:** The mRNA levels of FOXD1 (normalized and log2-transformed) were compared between HNSCC samples and normal counterparts in GSE6631 (**A**), GSE12452 (**B**), GSE25099(**C**), GSE30784(**D**)and TCGA-HNSC(**E**) datasets. The original data were retrieved from GEO database and TCGA, then plotted using Graphpad Prism 6.0 software. HNSCC, head neck squamous cell carcinoma; FOXD1, Forkhead box D1

Mounting study as well as pan-cancer analysis (**Supplementary Figure1,** downloaded from GEPIA2) have revealed that FOXD1 is overexpressed in a variety of cancers and has to do with unfavorable prognosis [11, 15]. Firstly, to investigate the FOXD1 mRNA expression in HNSCC, we mainly made use of GEO and TCGA databases to analysis the relevant information. Integration and analysis of the TCGA-HNSC cohort (502 cases) data showed that FOXD1 mRNA was obviously up-regulated in the TCGA-HNSC specimens compared with the normal counterpart (44 cases) using TCGA datasets (Fig. 1E). While as displayed in **Figure1 A-D**, data mining and questioning from GSE6631, GSE12452, GSE25099 and GSE30784 indicated that FOXD1 mRNA was significantly higher in HNSCC samples compared with the normal group, respectively. These data indicate that FOXD1 high expression may be related to the malignant regulation of HNSCC. The FOXD1 expression association among TCGA-HNSC subtypes was showed in **Supplementary Figure2**.

### Analysis of FOXD1 gene expression in HNSCC clinical samples and its clinical significance

**Fig. 2:**
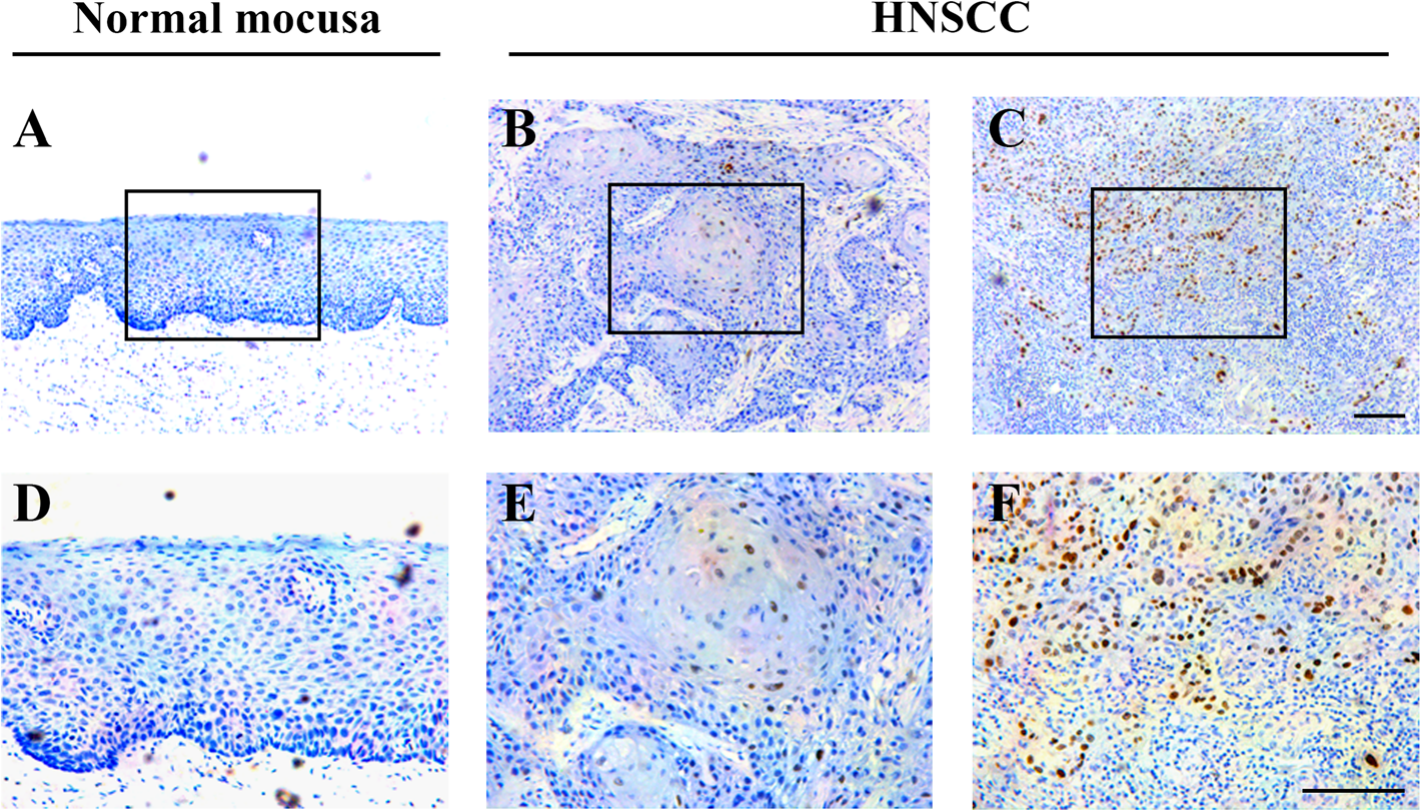
Nuclear location FOXD1 enriched in human HNSCC samples. **A, D:** Representative negative staining of FOXD1 in normal oral epithelial (100× A, 200× D); **B, E:** Representative low expression of FOXD in primary human HNSCC sample (100× B, 200× E); **C, F:** Representative high expression of FOXD1 in primary human HNSCC sample (100× C, 200× F). Scale bar: 100 μm

Next, to further reveal the expression pattern of FOXD1 in HNSCC clinical specimens as well as its relationship with clinicopathological parameters, we semi-quantitatively detected FOXD1 protein expression pattern in 110 patients with primary HNSCC by immunohistochemical staining. All clinical data of these patients are shown in Table 1 (49 females/61 males, average 61.49 years). The patient’s latest follow-up time ranged from 3 to 83 months (mean 42.4 months). As displayed in Fig. 2, FOXD1 showed a positive staining mainly in nucleus in HNSCC paraffin sections, whereas weak/negative staining was identified in the normal oral mucosa and the stroma of HNSCC. Based on our IHC-scoring system, FOXD1 expression in HNSCC/normal mucosa was classified. Thus, the expression of FOXD1 protein can be classified as high (*n* = 61) or low expression group (*n* = 49) in HNSCC clinical samples and negative (*n* = 8), high (*n* = 1) or low (n = 4) expression in normal HNSCC clinical specimens. Therefore, these data confirmed that the FOXD1 protein was highly expressed in HNSCC (*P* < 0.01, Fisher’s exact test). The correlation between FOXD1 expression and clinicopathological parameters were displayed in Table 1 (Chi-square test or Fisher’s exact test). It is clear that there were no obviously association between FOXD1 abundance and patients age, drinking and heavy tobacco usage, tumor size and pathological grading. Notably, FOXD1 expression was positively associated with cervical lymph nodes metastasis and clinical stage with *P* value 0.0218 and 0.0105 (Fisher’s exact test), respectively.

**Table 1:** Expression of FOXD1 and its associations with clinicopathological parameters in 110 patients with HNSCC

Clinicopathological parameters	Samples	FOXD1		***P*** values
		Low	High	
Gender		49	61	0.7019
Male	61	26	35	
Female	49	23	26	
Age				0.2463
≤ 60	48	18	30	
> 60	62	31	31	
Smoking				0.8382
No	74	32	42	
Yes	36	17	19	
Alcohol				0.3843
No	83	39	44	
Yes	27	10	17	
Tumor size				0.9766
T1–T2	72	32	40	
T3–T4	38	17	21	
Pathological grade				0.2074
I	78	38	40	
II–III	32	11	21	
Cervical node metastasis				**0.0218**
N(0)	58	32	26	
N(+)	52	17	35	
Clinical stage				**0.0105**
I–II	48	28	20	
III–IV	62	21	41	

Bold indicates statistical significance, with P values less than 0.05

### Aberrant overexpression of FOXD1 in HNSCC patients associated with poor outcome

**Fig. 3:**
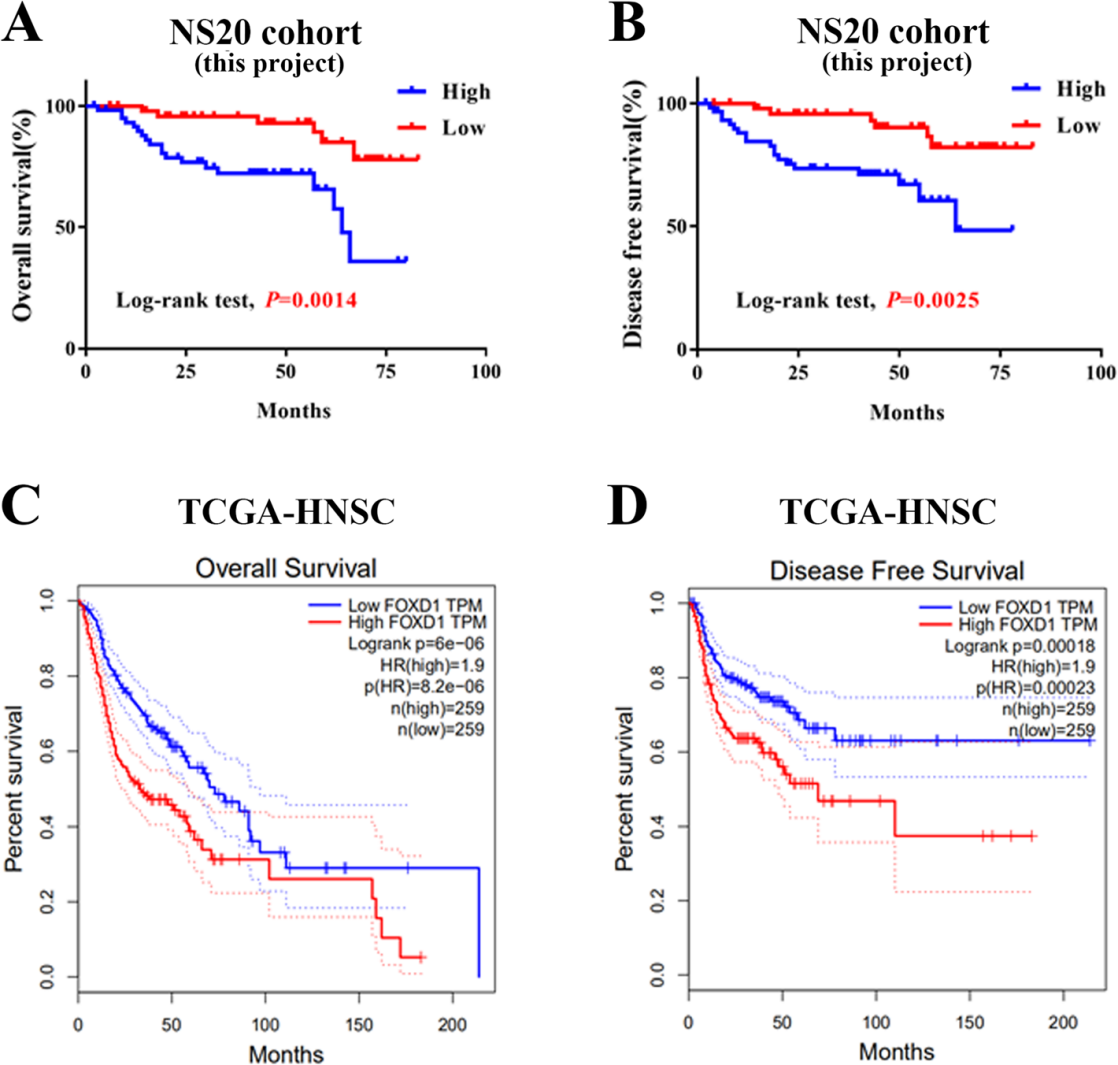
High FOXD1 expression positively associates with reduced overall survival rates in HNSCC patients. A**-A, B:** Overall survival (A) and disease-free survival (B) analyses of HNSCC patients with high or low expression of FOXD1 were estimated by IHC. **C, D:** Overall survival (C) and disease-free survival (D) plot of FOXD1 in TCGA-HNSC patients downloaded from cBioPortal(https://www.cbioportal.org/). Kaplan-Meier method with median cutoff and compared with Log-rank test

To explore prognostic significance of FOXD1 abundance in HNSCC patients, we analysis the relationship between its protein level and clinical prognostic. According to the latest follow-up data, 85 (77.28%) patients were still disease-freely alive, 8 (7.2%) survival with cervical nodal metastasis and/or local recurrences, whereas 25 (22.73%) patients died of post-surgical relapse, cancer metastases or other diseases. Further, as the Kaplan-Meier plots shown, patients with high FOXD1 abundance had obviously shorter overall-survival and disease-free survival than patients with low FOXD1 abundance (Log-rank, *P* = 0.0014, 0.0025, **Figure3 A, B**). Whereas, the similar conclusion form TCGA-HNSC cohort from GEPIA2 showed that the overall−/disease-free survival proportions in FOXD1^high^ groups was also significantly lower than those in FOXD1^low^ groups (Log-rank, *P* < 0.0001, 0.00018, Fig. 3C, D). To further evaluate the prognostic value of FOXD1 abundance in HNSCC, both univariate and multivariable survival analyses were executed by cox proportional hazards regression model. Univariate and multivariate cox analysis showed that high FOXD1 expression may be an independent prognostic factor for patient^’^s survival (as shown in Table 2.

**Table 2:** Univariate and multivariate survival analyses (proportional hazards method) for patients with primary HNSCC

Parameters	Univariate survival analysis			Multivariate survival analysis		
	Hazard ratio	95% CI	***P*** values	Hazard ratio	95% CI	***P*** values
Gender(male, female)	1.527	0.691–3.374	0.296	1.969	0.665–5.830	0.221
Smoking(Yes, No)	1.484	0.591–3.727	0.401	1.473	0.308–7.049	0.628
Alcohol use (Yes, No)	0.681	0.282–1.643	0.392	0.428	0.118–1.551	0.196
Age (> 60, ≤60)	0.682	0.311–1.496	0.340	0.529	0.224–1.246	0.145
Tumor size (T3-T4, T1-T2)	0.747	0.312–1.792	0.514	0.842	0.329–2.158	0.720
Pathological grade (II-III, I)	0.622	0.213–1.813	0.384	0.645	0.188–2.221	0.487
Cervical nodal metastasis (N+, N0)	1.175	0.536–2.578	0.687	0.881	0.359–2.157	0.781
Clinical stage (III-IV, I-II)	1.558	0.685–3.545	0.290	1.272	0.517–3.13	0.600
FOXD1 expression (high, low)	4.159	1.626–10.641	**0.003**	4.926	1.728–14.045	**0.003**

Bold indicates statistical significance, with P values less than 0.05

### FOXD1 knockdown inhibited migration and invasion, proliferation and facilitated apoptosis in HNSCC cells

**Fig. 4:**
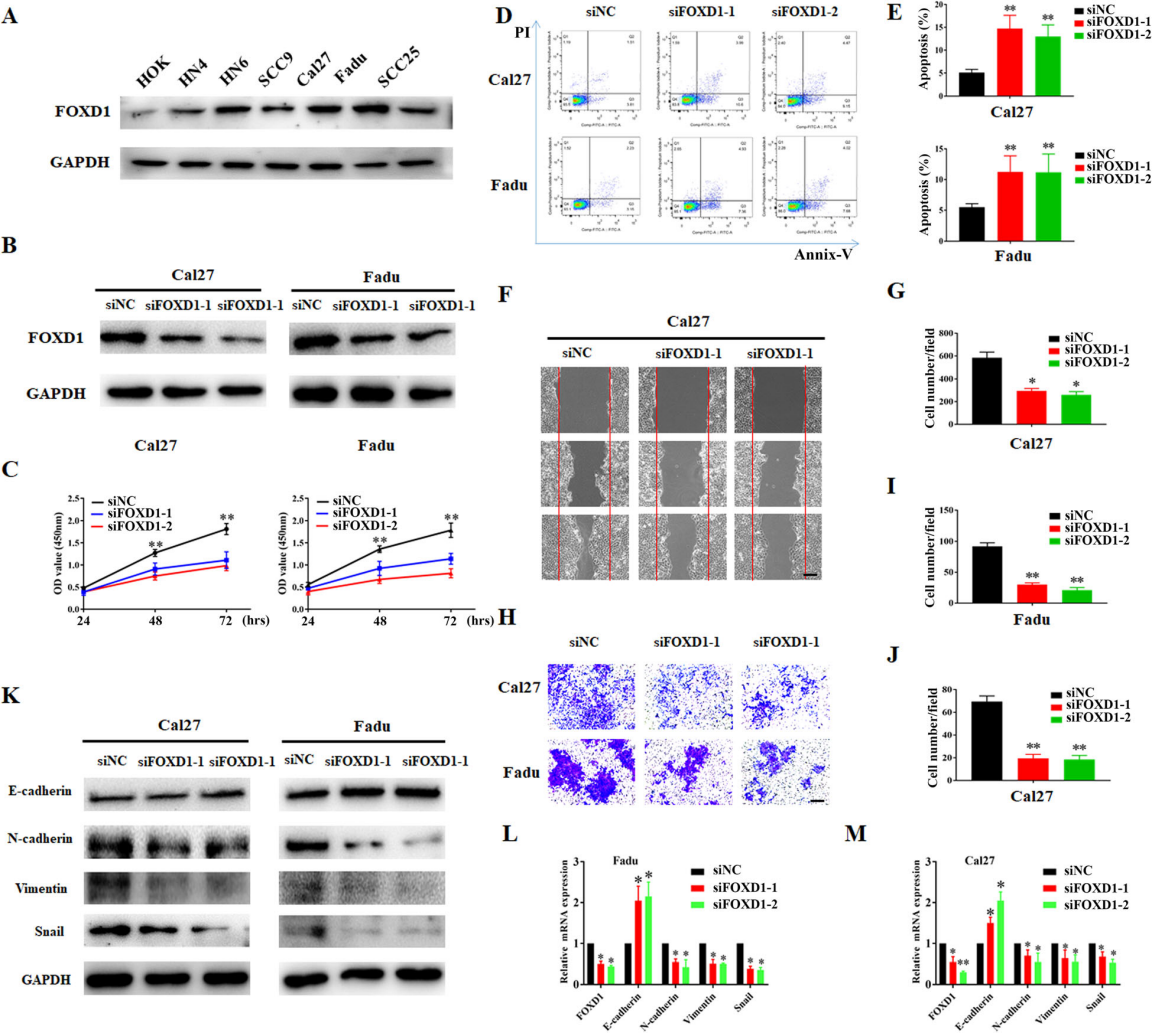
FOXD1 knockdown inhibits cell migration and invasioncell proliferation, cell migration and invasion and triggers apoptosis in HNSCC cells. **A:** Endogenous FOXD1 protein expression was measured in a panel of HNSCC cell lines as compared to normal human oral keratinocytes (HOK). **B:** Endogenous FOXD1 was efficiently silenced by two siRNAs (siFOXD1–1, siFOXD1–2) in FADU and Cal27 cells. Non-targeting siRNA was utilized as negative control (siNC). **C:** Cell proliferation was remarkably suppressed when endogenous FOXD1 was silenced as measured by CCK8 assays. **D, E:** Increased percentages of cell undergoing apoptosis were evident following FOXD1 knockdown as assayed by Annexin V-PI staining. **F, G:** Cell migration was determined in cells with FOXD1 knockdown by wound-healing assay in Cal27 cells. **H-G:** Cell invasion was determined in cells with FOXD1 knockdown by transwell invasion assay in Cal27 and Fdau cells. **K-M:** The protein and mRNA abundance of migration/invasion-relevant marker E-cadherin, N-cadherin, snail and Vimentin was compared in cells infected siFOXD1 or control siNC. Scale bar: 100 μm. Data shown from three independent experiments, *P < .05, **P < .01, Kruskall-Wallis test

To explore the cancer-promoting effects in HNSCC, we first  examined the FOXD1 abundance in several HNSCC cell lines and carried out the loss-of-function assay by siRNA modulated knockdown. As displayed in Fig. 4A, the expression of FOXD1 protein in all HNSCC cell lines was obviously higher than that in immortalized non-tumorigenic cells (HOK). Cal27 and Fadu with relatively higher expression of endogenous FOXD1 protein were selected for further knockdown assays. Two independent siRNAs targeting human FOXD1(siFOXD1–1, siFOXD1–2) were brought in cal27 and Fadu cells to detect the changes of FOXD1 abundance and cell phenotype. As shown in Fig. 4B, the expression of FOXD1 protein decreased significantly after transfection with siRNA, which confirmed the effectiveness of our gene knockdown method. Cell proliferation was obviously repressed in two type of cells after FOXD1 expression inhibited as decision by results analysis from CCK-8 experiments (Fig. 4C). Moreover, results from Annexin V-PI Flow cytometry experiment showed that the apoptosis rate of siFOXD1-treated cells obviously elevated from 4.9 to 14.2% in Fadu and from 5.1 to 15.6% in Cal27, respectively (Fig. 4D, E). The migration and invasion ability of FOXD1 knockdown cells were also examined by wound healing and Transwell experiments, respectively. FOXD1 knockdown obviously decreased the ability of cell migration (Fig. 4F, G) and invasion (Fig. 4H-J) in vitro. Consistent with these observed phenotypical varies following FOXD1 knockdown, the protein and mRNA expression of EMT/metastasis-associated marker Vimentin、N-cadherin and Snail were low-expression concomitant with E-cadherin high-expression (Fig. 4K-M). These results suggested that FOXD1 is involved in the regulation of the malignant phenotype of HNSCC and may be a valuable potential therapeutic target.

### Functional enrichment analysis of the differentially expressed genes between FOXD1 low/high groups

**Fig. 5:**
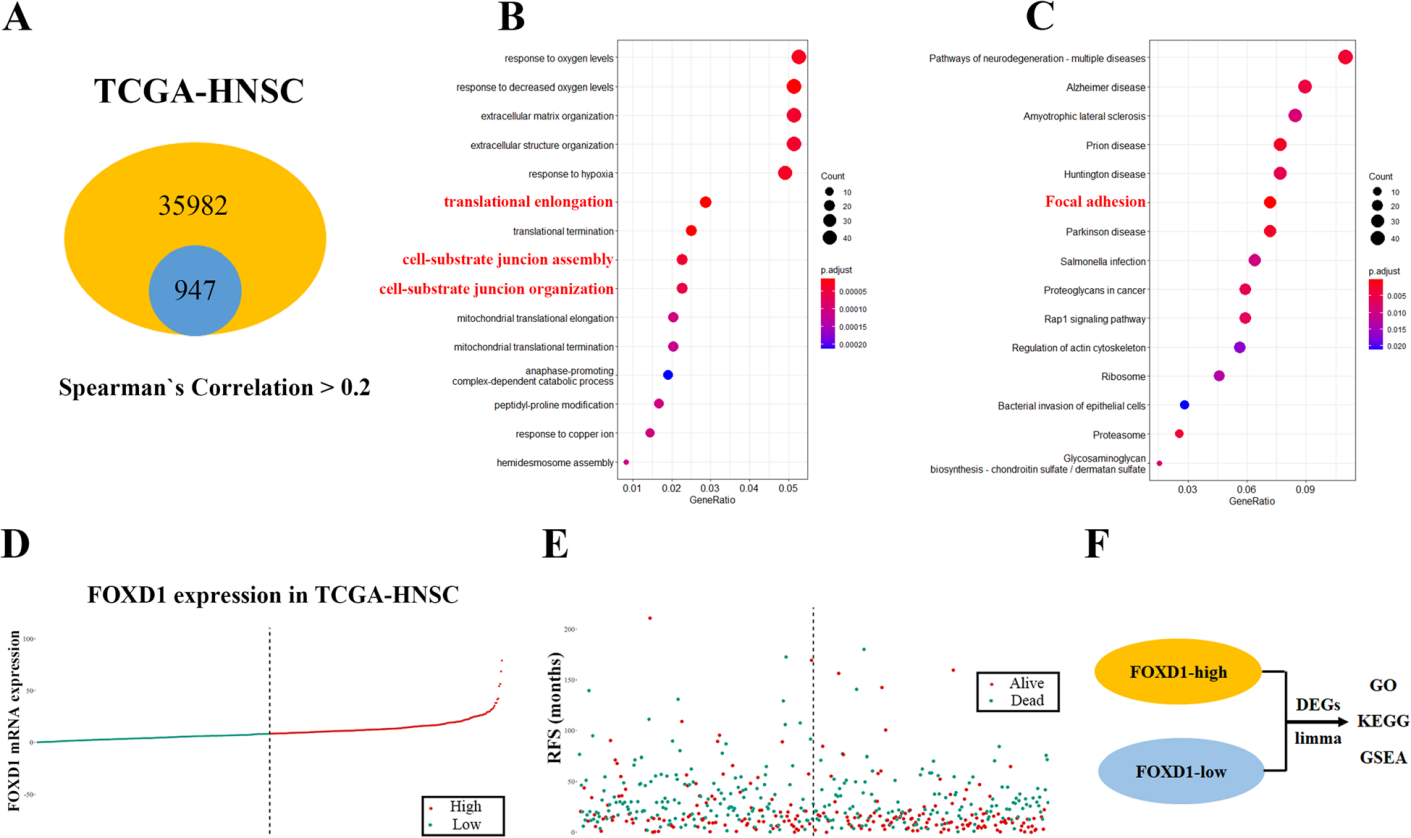
The biofunction of FOXD1 correlation genes. **A:** Schematic diagram of the numbers of FOXD1-associated-genes with Spearman’s correlation > 0.2 subset. **B, C:** The top 15 involved significant biological process (GO, B) and pathways (KEGG, C) in these positive correlation genes. **D-E:** Scatter plot (D) displaying the FOXD1 level of each patient from TCGA-HNSC dataset. According to the median-cutoff FOXD1 expression, patients were divided into high- or low-expression groups and survival status plot (E) shows the patients with the high- or low- group. **F:** Schematic diagram of further bioinformatic analysis between high- or low- group

**Fig. 6:**
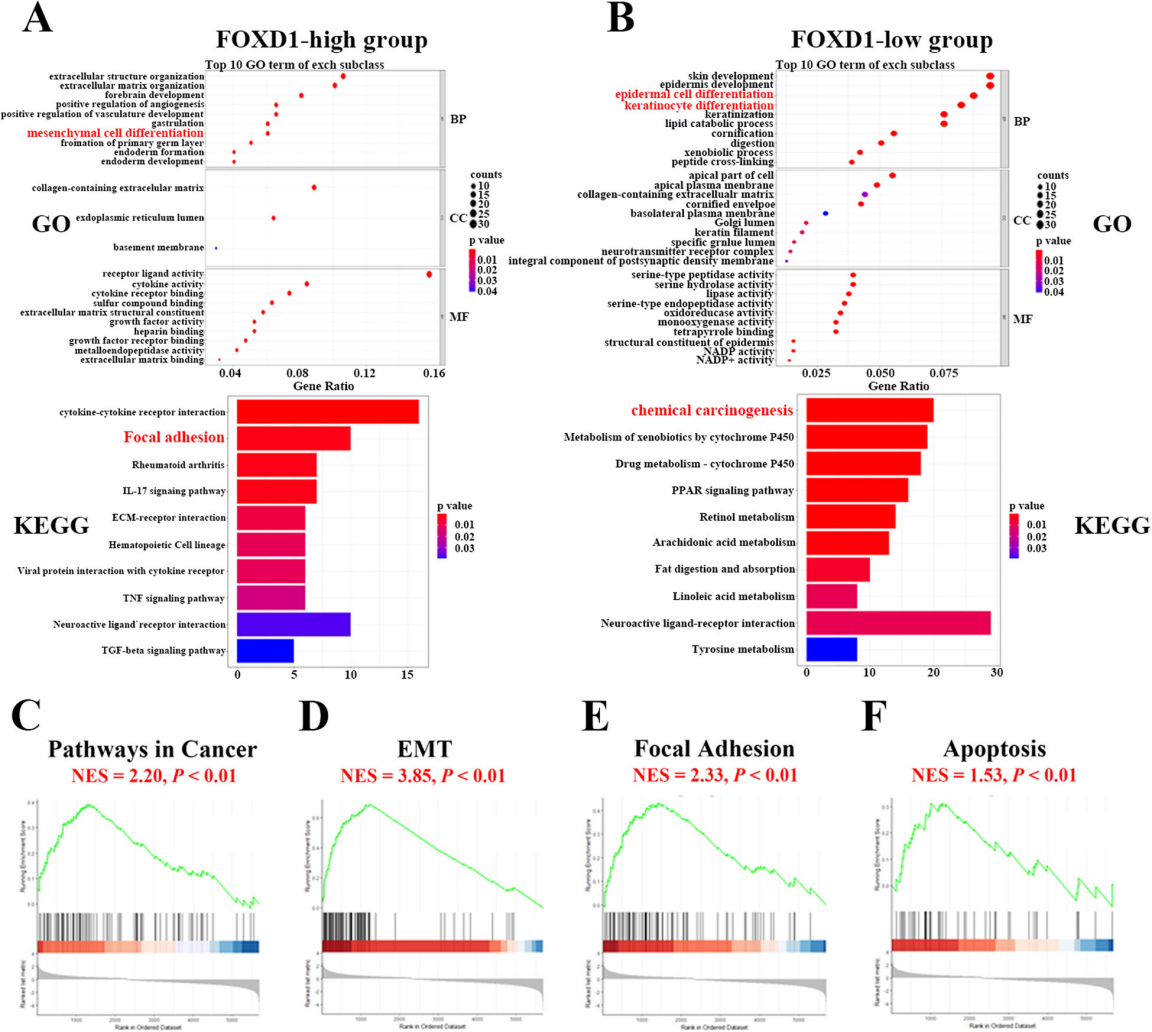
The GO, KEGG and GSEA analysis results for the differentially expressed genes between high-FOXD1 and Low-FOXD1 groups. **A:** GO (upper panel) and KEGG (low panel) analysis for the genes in high-risk group. **B:** GO (upper panel) and KEGG (low panel) analysis for the genes in low-risk group.**C-F:** The most involved significant pathways via GSEA

In order to complement the in vitro loss-of-function experiment in investigating FOXD1 pro-tumorigenic functions. Firstly, we downloaded the FOXD1 correlated-gene chart from TCGA-HNSC on GEPIA2, filtered the correlation score > 0.2 geneset (Fig. 5A) and performing gene ontology (GO) and Kyoto Encyclopedia of Genes and Genomes (KEGG) analysis, and the data suggests that FOXD1-positive-corrlated genes enriched in numerous cancer related biofunction and pathways such as cell-substrate junction and focal adhesion(Fig. 5B, C). Then, we further used TCGA-HNSC data for bioinformatics analysis to recognize the nominee genes that might be potentially associated with FOXD1, the expression of which was divided into high-expression group and low-expression group (median-cutoff) by the method of bipartition in TCGA-HNSC data and then were in thrall to GO and KEGG and Gene Set Enrichment Analysis (GSEA) analyses (Fig. 5D-F). GO and KEGG analysis the up-regulated genes in high-risk group associated with FOXD1 were significantly enriched in Focal adhesion, Cytokine-cytokine receptor interaction, IL-17 signaling pathway, ECM-receptor interaction, TNF signaling pathway, TGF-beta signaling (Fig. 6A). On the contrary, KEGG and GO analysis for the down-regulated genes associated with FOXD1 in Low-risk group were related to apoptotic process, metabolism (Fig. 6B). GSEA analysis revealed that differentially expressed genes between high-FOXD1 and Low-FOXD1 groups were involved significant pathways included KEGG Pathways in cancer (Fig. 6C), EMT pathway (Fig. 6D), KEGG Focal adhesion (Fig. 6E) and Apoptosis (Fig. 6F). In conclusion, combined with cell assay in vitro and bioinformatics results extremely support the idea that FOXD1 is an newly oncogene in HNSCC.

## Discussion

As a core member of the human FOX gene family, FOXD1 expression may be an oncogene involving cancer progression and has been critically participated in cell migration and apoptosis, cancer stem cell self-renewal as well as other physiological and pathological processes [16–18]. Previous studies have shown that FOXD1 may be an oncogene related to tumor development and act as a potential diagnostic biomarker and anti-cancer therapeutic target [9, 11, 19]. Herein, this study revealed the FOXD1 expression pattern in HNSCC, uncovered its prognostic and clinicopathological significance and also revealed its the oncogenic roles of FOXD1 by the loss-of function method and bio-informatics analysis. Our results together highly suggested that as a newly hypothesized oncogene FOXD1 promotes HNSCC development and also a novel biomarker with clinical translation potential.

HNSCC initiation and development are attributed through consecutive histopathological stages from normal epithelial cells to squamous-cell carcinoma cells which is mainly due to the activation of genetic predisposition, oncogenes and the inactivation of tumor suppressor genes [20–22]. In particular, dysregulation of the FOX family have been proved to be involved in nearly all stages of HNSCC tumorigenesis [23]. Among these FOX gene regulators, FOXD1 has been increasingly recognized as a key agent for cancer initiation, unrestricted growth, and metastasis, and has been proven to be a promising therapeutic target [17].

Here, we found that FOXD1 is significantly elevated in most HNSCC specimens as proofed by obviously overexpressed FOXD1 mRNA in GEO and TCGA datasets as well as up-regulation of FOXD1 protein in our HNSCC patients cohort. To our knowledge, this is the first study to confirm an abnormal pattern of FOXD1 overexpression in HNSCC. Previous findings have shown the clinical significance of FOXD1 abundance in various human cancers. For instance, elevated FOXD1 expression is obviously associated with lymph nodes metastasis, tumor size and advanced stages in non-small cell lung cancer [15], nasopharyngeal carcinoma and colorectal cancer [23, 24]. We further established overexpression pattern of FOXD1 and shown that its overexpression significantly associated with lymph node metastasis and clinical stage in HNSCC, while there was no significant correlation between FOXD1 expression and other clinicopathologic parameters. Moreover, up-regulation of FOXD1 was obviously associated with the decrease of survival rate via Kaplan-Meier survival data analysis, thus suggesting that FOXD1 might be a valuable prognostic biomarker for HNSCC. Therefore, Multivariate survival analysis using cox proportional hazards regression model results confirmed that FOXD1 expression was an independent prognostic factor for HNSCC. This was consistent with our previous research results in OSCC [16] and other associated OSCC research [22, 25]. Accumulating evidence has revealed that FOXD1 is vitally participated in tumorigenesis by facilitating cell migration, invasion, proliferation, angiogenesis and inhibiting apoptosis [9, 26]. Consistent with this, our in vitro loss-of-function experiment results indicate that FOXD1 knockdown leads to a diminished in proliferation, migration and invasion in HNSCC cells. Additionally, bioinformatics analysis results further indicated that FOXD1 was involved significant pathways included KEGG Pathways in cancer, EMT, Focal adhesion, Apoptosis, TGF-beta signaling, Cell cycle etc. Consistently, previous studies have revealed that FOXD1 facilitates cell proliferation and inhibits apoptosis by promoting the expression of PIK2 in colorectal cancer [24]. Fengping Pan and his colleagues revealed that FOXD1 promoted CRC cell malignant phenotypes by activation of the ERK 1/2 signaling pathway [6]. Complementary, in melanoma cancer, FOXD1 was critically involved in invasion and migration via indirect regulation of RAC1b and MMP-9 alternative splicing in melanoma cells [8]. Of note, the inhibition of FOXD1 expression affected cell related malignant phenotypes such as cell proliferation, cell migration and invasiveness, cell apoptosis and ultimately leads to tumor regression and reduction of tumor metastasis. Taken together, combined with bio-informatics analysis and the results of our findings and others strongly favors the notion that FOXD1 probably functions as a hypothetical oncogene by facilitating cancer cell proliferation, migration and invasion. Suppression of FOXD1 expression via genetic method might hold translational promise in the treatment of HNSCC.

## Conclusion

In conclusion, our all results indicate that a subgroup of HNSCC patients who FOXD1 aberrantly up-regulation in an important and reveals its pro-oncogenic role in promoting the occurrence and development of HNSCC. However, there is still a large gap between the role of FOXD1 in the development of HNSCC and the effective treatment of FOXD1.

## Supplementary Information

**Additional file 1 Supplementary Figure1. FOXD1 mRNA expression pattern in pan-cancer analysis**.

**Additional file 2 Supplementary Figure2. FOXD1 mRNA expression pattern in TCGA-HNSCC subtypes. A-F:** The mRNA levels of FOXD1 (normalized and log2-transformed) from TCGA-HNSCC datasets were compared between clinical subtypes: Clinical stage(I- IV, **A**), Pathological grade(I-III, **B**), HPV infection status(**C**), Tumor size(T1-T4, **D**), Cervical nodal metastasis(**E**), Distant metastasis(**F**). Student’s *t* test or Kruskall-Wallis test.

## Data Availability

The dataset used during the present study is available from the corresponding author upon a reasonable request.
