# Supplementary figures and images for: Aberrant overexpression of transcription factor Forkhead box D1 predicts poor prognosis and promotes cancer progression in HNSCC
Source: BMC Cancer. 2021 Nov 12;21:1205. doi: 10.1186/s12885-021-08868-4 (PMC8588630; doi:10.1186/s12885-021-08868-4)

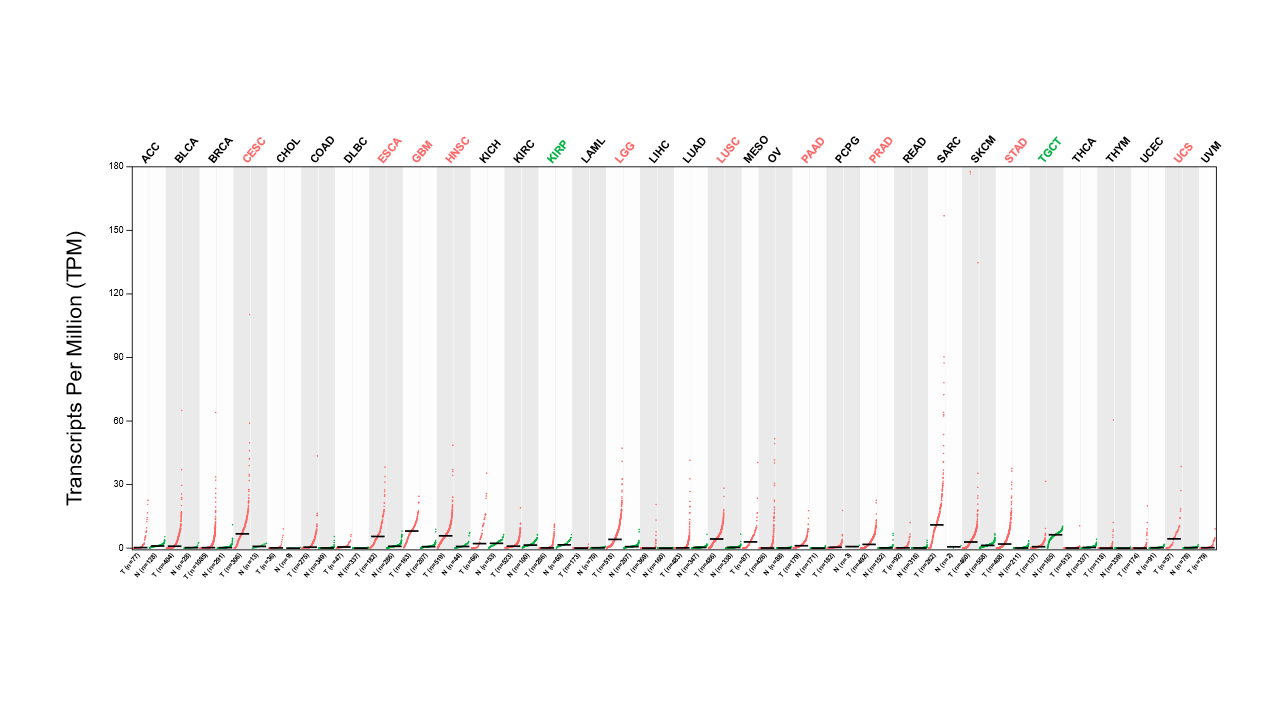

Supplement: Supplementary file 1 — Additional file 1 Supplementary Figure1. FOXD1 mRNA expression pattern in pan-cancer analysis. [file 12885_2021_8868_MOESM1_ESM.tif]

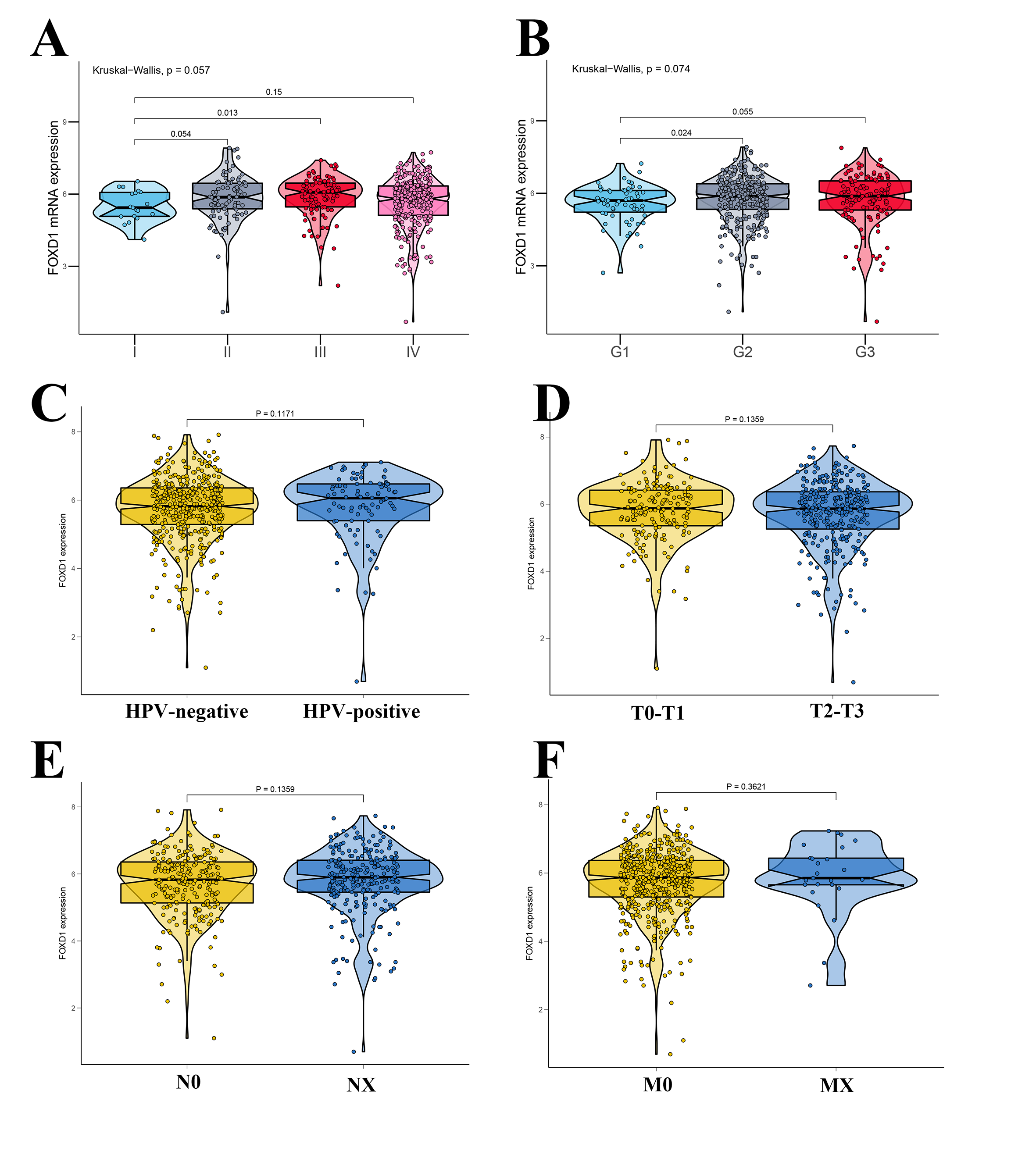

Supplement: Supplementary file 2 — Additional file 2 Supplementary Figure2. FOXD1 mRNA expression pattern in TCGA-HNSCC subtypes. A-F: The mRNA levels of FOXD1 (normalized and log2-transformed) from TCGA-HNSCC datasets were compared between clinical subtypes: Clinical stage(I- IV, A), Pathological grade(I-III, B), HPV infection status(C), Tumor size(T1-T4, D), Cervical nodal metastasis(E), Distant metastasis(F). Student’s t test or Kruskall-Wallis test. [file 12885_2021_8868_MOESM2_ESM.tif]
